# Concomitant ectopic *Enterobius vermicularis* infection in uterine cervical cancer

**DOI:** 10.1186/s12905-024-03073-4

**Published:** 2024-04-27

**Authors:** Iztok Takač, Rajko Kavalar, Matija Rudolf Lovrec, Vida Gavrić Lovrec

**Affiliations:** 1 Division for Gynecology and Perinatology, University Medical Centre Maribor, Ljubljanska ul. 5, Maribor, 2000 Slovenia; 2 Department of Obstetrics and Gynecology, Faculty of Medicine, University of Maribor, Maribor, 2000 Slovenia; 3 Department of Pathology, University Medical Centre Maribor, Maribor, 2000 Slovenia

**Keywords:** *Enterobius vermicularis*, Extraintestinal manifestation, Female genital tract, Uterine cervix, Cervical cancer

## Abstract

**Background:**

*Enterobius vermicularis* (*E. vermicularis*), also referred to as pinworm, is a widespread human intestinal parasite which predominantly occurs in young children, making their caretakers a population at risk for the transmission of this helminth. It can occasionally affect extraintestinal organs and tissues, including the female genital tract. Infestation can be asymptomatic or manifest as different kinds of gynaecological disorders, such as pelvic inflammation mimicking tumours, abnormal uterine bleeding, or vaginitis. Diagnosis is made by identifying ova in the sample collected from the perineal skin using a transparent adhesive tape or microscopic examination of resected tissue. Mebendazole is the first-line medication and should also be administered to all household members.

**Case presentation:**

We present a case of a patient who had undergone surgery for invasive cervical cancer with an accidental finding of *E. vermicularis* eggs in the cervix.

**Conclusions:**

Although not very common, infestation with *E. vermicularis* should be considered in differential diagnoses of various gynaecological disorders accompanied by histological findings of granulomatous inflammation.

## Background

*E. vermicularis*, also known as pinworm, is the most prevalent intestinal parasite, and enterobiasis affects over 200 million people worldwide [1]. Humans are the only known hosts of this parasitic worm, as its entire life cycle occurs within the lumen of the human gastrointestinal tract [2].

Occasionally, extraintestinal organs and tissues can be infested by *E. vermicularis*, including the female genital tract [3]. Several case reports present different clinical manifestations; however, according to publications, *E. vermicularis* in the uterine cervix is rare [4]. Until now, no report of *E. vermicularis* infestation of the cervix with concomitant cervical cancer was recorded.

We present a case of a patient who had undergone surgery for invasive cervical cancer with an accidental finding of *E. vermicularis* eggs in the cervix.

## Case presentation

A 35-year-old patient was admitted to the Department of Gynecology at the University Medical Centre Maribor, for surgical treatment of verified cervical squamous cell carcinoma FIGO stage IB, grade 2 without necrosis. A PAP smear of the cervix revealed a high-grade squamous intraepithelial lesion (HSIL). Histologic examination of two colposcopically suspicious regions confirmed invasive squamous cell carcinoma FIGO stage IB, grade 2, with necrosis and without lymphovascular invasion (LVI) or perineural invasion (PNI). Clinical examination, ultrasonography and MRI failed to show any cervical tumour or lymph nodes in the lower pelvis. Tumour marker squamous cell carcinoma antigen (SCC) was slightly elevated (1,7 ng/mL). A differential blood count was not performed; there was no information about potential eosinophilia. The patient was healthy, without any accompanying diseases. She had regular menstrual cycles; she gave birth twice and had one ectopic pregnancy. She worked as a teacher in a kindergarten. There was no record of recent travels or prior anthelminthic treatment.

**Fig. 1:**
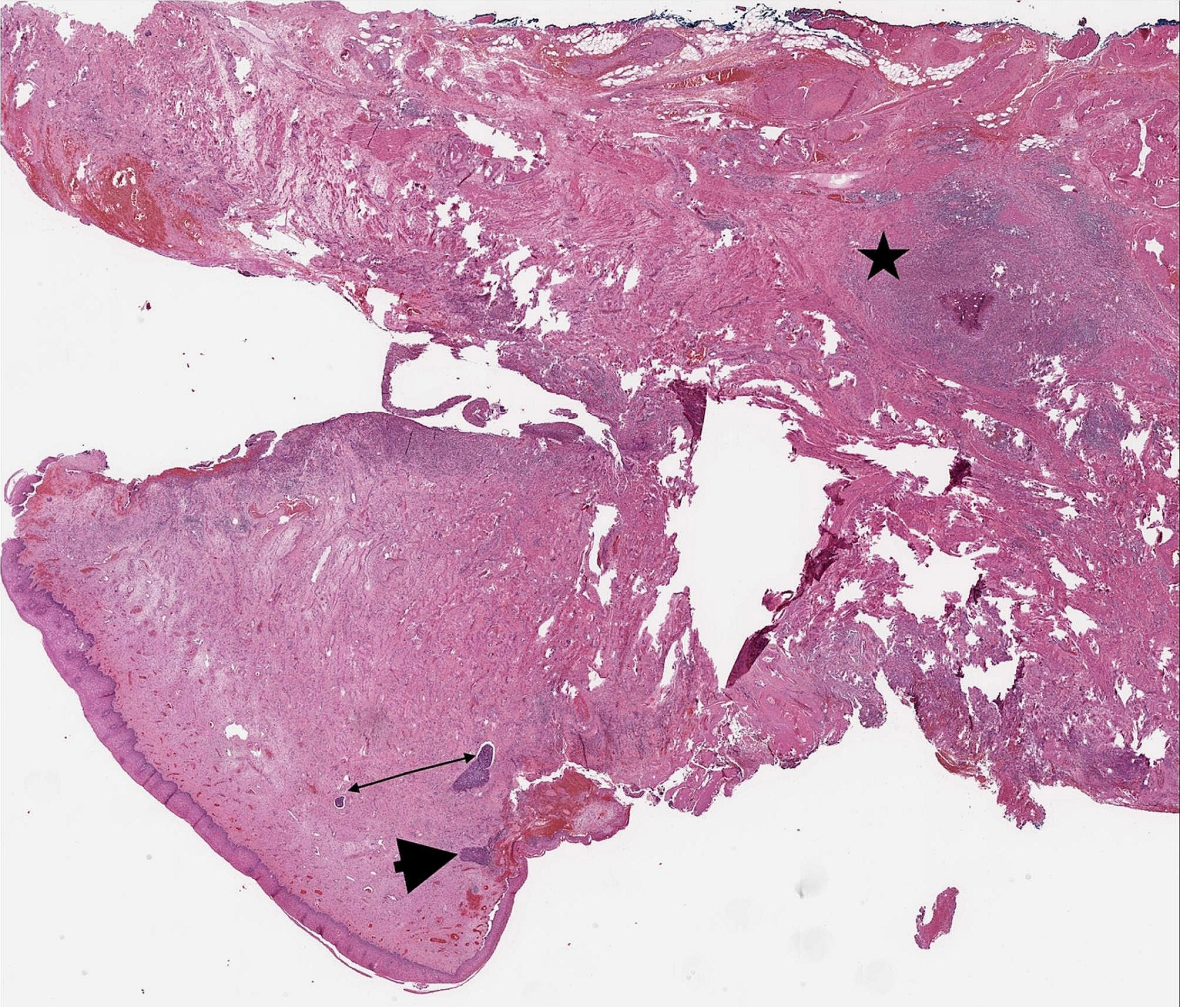
Residua of an invasive nonkeratinizing squamous cell cancer in the cervix (arrowhead), partly ulcerated and with lymphovascular invasion (double arrow). Deep in the cervical stroma away from the SCC, foci of acute and chronic inflammation with foreign body granuloma formation are present and, in their center, there are multiple *Enterobius vermicularis* eggs (star). (H&E, original magnification x2)

**Fig. 2:**
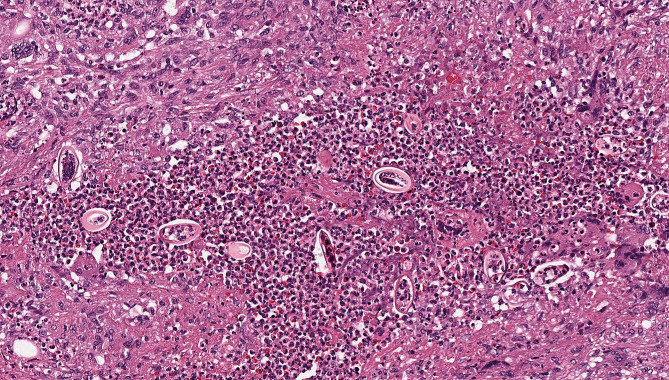
The center of granuloma with dense eosinophilic and neutrophilic granulocytes with parasitic eggs. On the edge of granuloma there are some multinucleated foreign body giant cells. No pinworm larvae are present. (H&E, original magnification x20)

Diagnostic cold knife conisation was performed a month later to determine the actual stage of the disease. Histological findings were that of an invasive squamous cell carcinoma with a diameter of 20 mm and a depth of 15 mm, with LVI but without PNI and invasion in blood vessels. According to FIGO Classification from 2019, the disease was diagnosed as stage FIGO IB2 and radical surgery sec. Wertheim-Meigs-Novak, including radical hysterectomy, bilateral salpingectomy and pelvic lymphadenectomy were performed. Final pathological findings confirmed residual 4,5 mm thick invasive squamous cell carcinoma of the cervix without contact with the resection plane, with LVI, non-keratinizing, p-16 positive. Twenty-nine lymph nodes were removed, and all were cancer cell free. At the microscopic examination of the resected uterus, the residua of an invasive nonkeratinizing squamous cell cancer (SCC) were present in the cervix, partly ulcerated and with lymphovascular invasion. Deep in the cervical stroma away from the SCC, foci of acute and chronic inflammation with foreign body granuloma formation were present. In their center, there were multiple *E. vermicularis* eggs (Fig. 1). The eggs, identifiable by the dimensions and form as eggs of *E. vermicularis*, were oval with one flattened side, some altered by the regressive process. They were measuring approximately an average of 50–60 µ in length and 20–25 µ in width. No pinworm larvae were present (Fig. 2). Diagnosis of *E. vermicularis* infection was confirmed by the Laboratory for Parasitology of the Institute of Microbiology and Immunology, Faculty of Medicine, University of Ljubljana. In the postoperative period, the patient complained of paraesthesia in the legs and hypaesthesia in the thighs and had a transient elevation of liver enzymes. After the diagnosis of *E. vermicularis* infestation was made, mebendazole was administered. Ten days after surgery, the patient was released from the hospital in a stable state. Mebendazole was also administered to all family members. The patient complained about occasional urinary incontinence, and six months after the surgery, a vesicovaginal fistula was diagnosed. It was successfully surgically corrected. No signs of disease were discovered at 6-month check-ups over two years.

Written consent was obtained from the patient, allowing the processing of her medical and personal data for purposes other than medical care.

## Discussion

*E. vermicularis* is predominantly an intestinal parasite. The infective cycle begins with the ingestion of parasitic ova, transferred by the fingers from perineal skin to the mouth or nose (autoinfection) or various objects, such as furniture, door handles, banknotes, etc. [2]. Other people, most commonly family members and kindergarten or school children can be infected from there [5]. This was probably the way of infection in our patient who worked in the kindergarten. A recent study in Slovenia showed that the overall prevalence of *E. vermicularis* infestation in symptomatic preschool and school children was 34.2%, and the mean age of children positive for *E. vermicularis* was 5.8 years. Family size affected the rate, as the mean number of siblings was higher in positive children [5]. The infection predominantly occurs in young children, ages 5–10 years, and is more frequent in temperate regions of Western Europe and North America. Factors such as overcrowding and poor sanitation also increase the risk of infection [2].

After ingesting the ova, larvae are released into the small intestine, typically the duodenum. They mature as they migrate towards their mating site in the caecum and its vicinity. This process lasts 2–6 weeks. After mating, male worms die, and fertilised females migrate at night through the anus to the warm and moist skin of perineal folds, where they lay up to 15,000 ova. This movement of the pinworms, along with the gelatinous substance deposited with the eggs, causes perineal itching and scratching, resulting in finger contamination, thus completing the cycle [2, 3].

*E. vermicularis* infection rarely involves extraintestinal sites, with the female genital tract being the most reported [6]. Recently, liver involvement was described and attributed to hematogenous migration in the setting of phlebitis with thrombosis of the inferior mesenteric vein [7]. Infestation of the eye and nasal mucosa have been described, and urinary tract infestation leading to recurrent urinary tract infections [8–10].

Genital infestation may result from the migration of gravid female worms from the perianal area to the vagina [11]. There are reports in the literature about ovarian enterobiasis, presenting as tubo-ovarian abscess with peritonitis [12], salpingitis with oophoritis, dermoid [3] or peritoneal lesions suspected to be malignant disease or metastases of ovarian cancer [6, 13]. Infestation of the uterus can be asymptomatic [14], and the presence of *E. vermicularis* can be detected accidentally. On the other hand, *E. vermicularis* infection can also affect the endometrium and cause abnormal uterine bleeding [15] or postmenopausal bleeding [16]. Infection of the endometrium might also cause fertility problems [17]. *E. vermicularis* ova were discovered in a cervical PAP smear, but according to our knowledge, not yet in the cervix of a patient with cervical cancer. Cervical infestation can cause a wide range of symptoms or be asymptomatic [4, 11].

Enterobiasis is usually diagnosed via microscopic identification of ova in the sample collected from the perineal skin by a transparent adhesive tape. This method is cheap, easy to perform and ensures a rapid result [1]. A stool sample is usually not helpful for a diagnosis since only 5% of those infected have the ova in stool, and adult worms are generally not passed in the faeces [2]. On microscopic examination of the tissue, parasitic ova and adult pinworms are often associated with acute and chronic inflammation with foreign body granuloma formation [2].

*E. vermicularis* infection should be treated with anthelminthic agents after its conformation [1]. In Slovenia, we use mebendazole or albendazole equivalently. A single dose of the drug is administered and then repeated after 14 days. Because *E. vermicularis* is easily transmitted among family members, simultaneous treatment of household members is recommended [18].

## Conclusions

To our knowledge, this is the first case of *E. vermicularis* diagnosed in the cervical stroma of a patient with cervical cancer. Although not very common, infestation with *E. vermicularis* should be considered in differential diagnoses of various gynaecological disorders accompanied by histological findings of granulomatous inflammation.

## Data Availability

All data generated or analysed during this study are included in this published article.

## Abbreviations

HSIL: High–grade squamous intraepithelial lesion
LVI: Lymphovascular invasion
PNI: Perineural invasion
SCC antigen: Squamous cell carcinoma antigen
